# Calibrating laser Doppler anemometers utilizing an optical chopper

**DOI:** 10.1088/1681-7575/adac64

**Published:** 2025

**Authors:** Christopher J Crowley, Iosif I Shinder, Michael R Moldover, Joey T Boyd, B James Filla, Aaron N Johnson

**Affiliations:** National Institute of Standards and Technology, Gaithersburg, MD 20899, United States of America

**Keywords:** laser Doppler anemometer, laser Doppler velocimeter, airspeed measurements, airspeed standard

## Abstract

Laser Doppler anemometers (LDAs) use scattered light to determine velocity components of a flowing fluid. The operating principal of LDAs is simple conceptually; however, it is impractical to trace the LDA-determined velocities to the SI by characterizing the LDA’s subsystems that generate, detect, and process optical signals because these subsystems are complex and include proprietary features. To circumvent this, we calibrated the complete LDA systems utilizing an optical chopper blade as an accurate, SI-traceable velocity standard. The calibrations achieved the expanded velocity uncertainty 0.094% at a 95% confidence level. We calibrated two LDAs that differed in manufacturer, focal length (in the ratio 3.3:1), sensing volume (in the ratio 100:1), and orientation (vertical and horizontal bisectors of the LDA’s crossing beams). To compare the calibrations, we measured airspeeds in NIST’s wind tunnel using both LDAs. The results differed from each other by, at most, 0.2% throughout the airspeed range (0.5–30) m s^−1^.

## 1. Introduction

A laser Doppler anemometer (LDA) is a stable, non-intrusive measurement system that is well suited for measuring airspeed in a wind tunnel [1, 2]. Modern LDAs measure the speed of a tracer particle passing through the optical interference pattern formed by the intersection of two focused laser beams originating from the same coherent source [3]. For intersecting beams with spherical symmetry (e.g. plane wave beams or Gaussian beams intersecting at their focal points), the fringes are nearly uniform parallel planes. When the flow sweeps a tracer particle through the fringes, the particle scatters light at the frequency determined by the particle’s velocity component perpendicular to the fringes ($V_{⊥}$) divided by the fringe spacing. In principle, knowledge of the wavelength of the laser beams and their angle of intersection is sufficient to use an LDA to measure $V_{⊥}$ without calibration. However, doing this was difficult in practice, so we instead calibrated the LDA against an SI-traceable velocity generated by an optical chopper to achieve the low relative uncertainties $U_{r}$ in this work [2, 4, 5]. Here, $U_{r}\left(V_{⊥}\right)=0.094\%$ for $0.5\;m\cdot {\;s}^{-1}⩽V_{⊥}⩽30\;m\cdot {\;s}^{-1}$at 95% confidence level^1^.

Several factors make LDA calibrations more complicated than calculating the fringe spacing. For example, slight misalignments of the optics prevent the LDA’s beams from intersecting at their focal point [6]. It is possible to calculate the effects of misalignment [7] and/or improve the alignment [8, 9]; however, it is difficult to quantify the optical path accurately enough to trust the calculations at the present uncertainty level $U_{r}\left(V_{⊥}\right)=0.094\%$. Direct imaging of the fringe pattern would provide all the optical information needed [10]; however, many LDAs apply a frequency shift to one of the beams meaning the imaging device must track a fringe pattern that is modulated at a frequency on order of 10 MHz for these LDAs.

In addition to the complexity of an imperfect fringe pattern, an LDA calibration must account for the complex processing of the light scattered by tracer particles. Typically, the scattered light is detected by a photomultiplier and the photomultiplier signals are processed by a burst spectrum analyzer (BSA). The BSA Fourier transforms the signals, filters them, fits them to functional forms to obtain values of $V_{⊥}$ that account for the diverse trajectories taken by tracer particles through the fringe pattern. The hardware and the algorithms in the BSA are sophisticated and proprietary, making the traceability of the LDA outputs to SI velocity components intractable.

**Figure 1.**
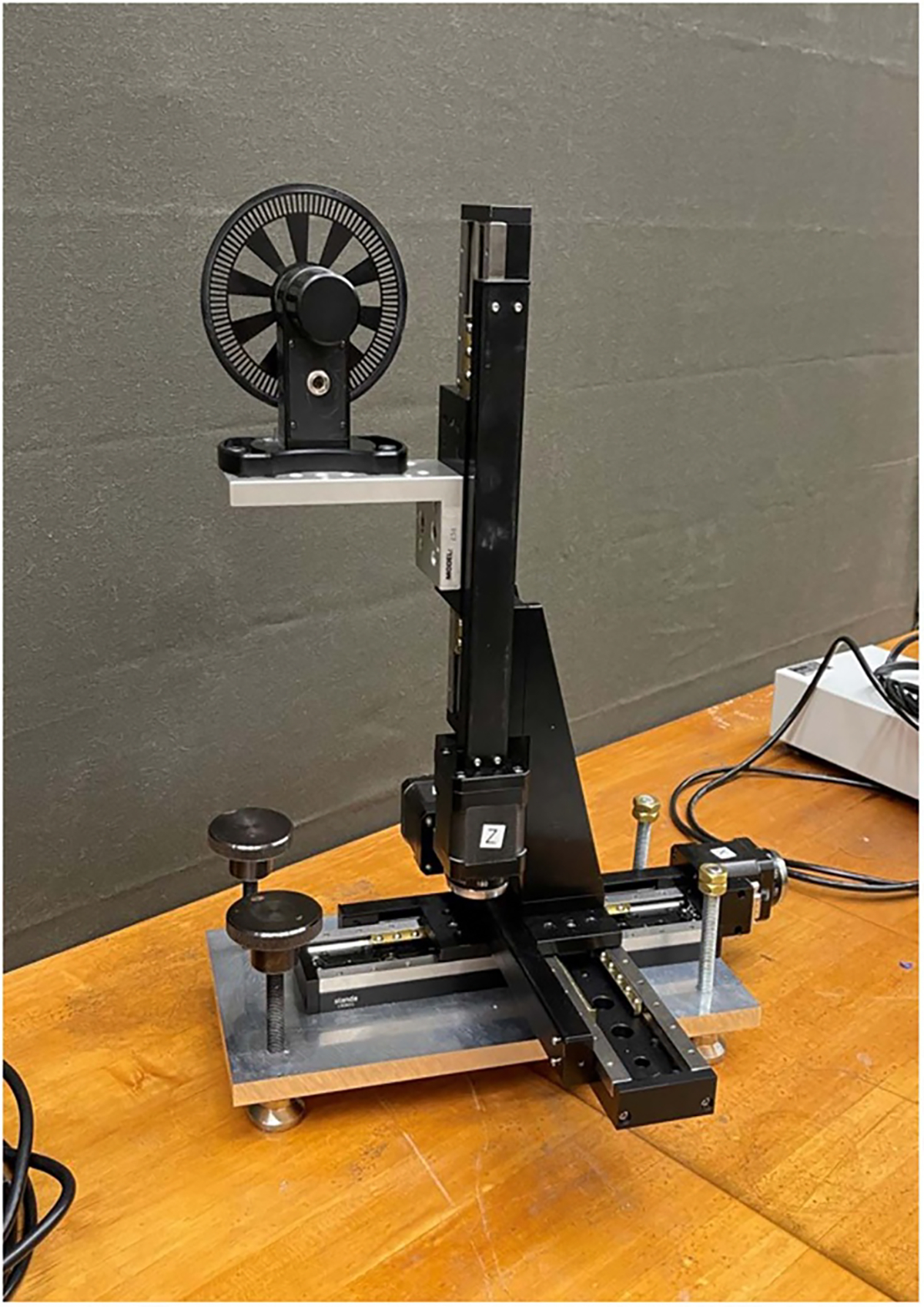
Chopper mounted on traversing stages. The chopper was not used to chop light beams, but only to move its outer rim at a known velocity.

**Figure 2.**
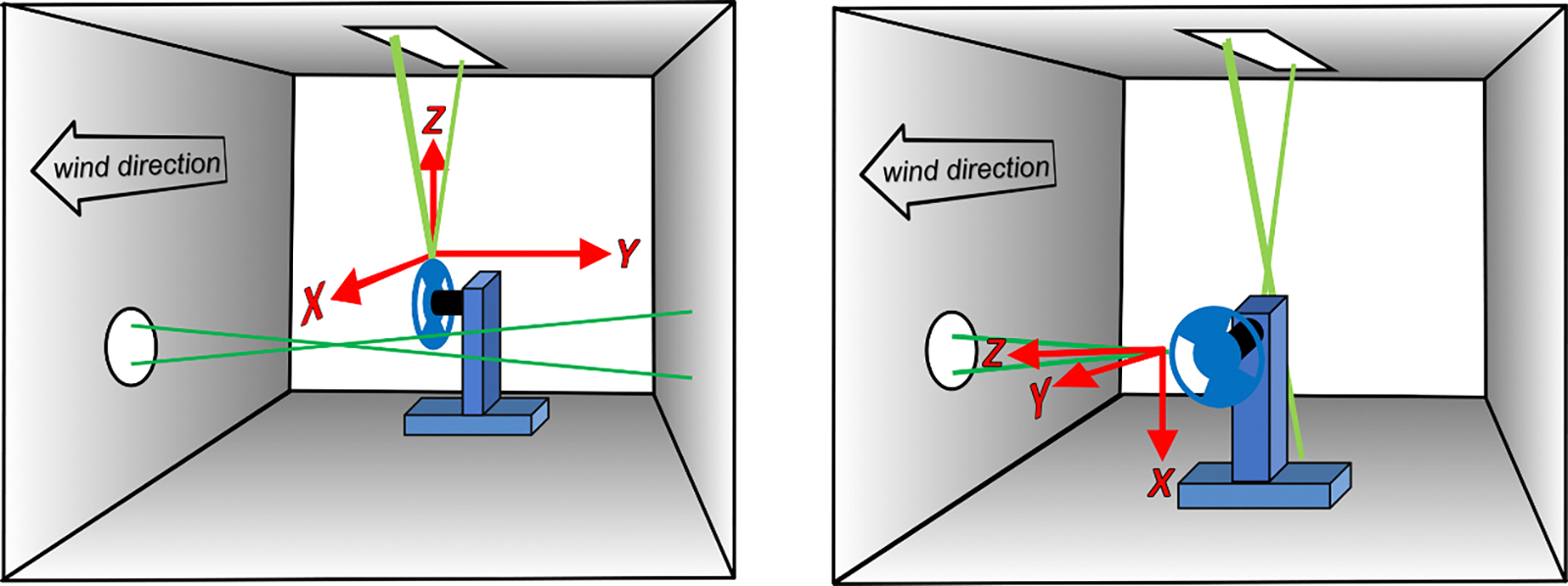
Orientation of the chopper during *in situ* calibrations of the LDAs. Left: calibration of ${LDA}_{\text{roof}}$ while it was installed atop the tunnel’s roof. Right: calibration of ${LDA}_{\text{wall}}$ while it was installed on the outer side of tunnel’s wall. Note: after calibration, ${LDA}_{\text{wall}}$ must be rotated 90° about its horizontal axis so that flows in wind direction are in the plane formed by ${LDA}_{\text{wall}}$’s laser beams.

To circumvent these complexities, we treat the LDA and its BSA as a ‘black-box’ and perform, an end-to-end calibration of the LDA system. For a calibrated velocity standard, we fastened a 3 mm wide acetal plastic strip to the rim of a commercially-manufactured optical chopper. The chopper’s angular velocity was measured using the built-in photodiode and an SI-traceable frequency counter. The average diameter of the rim was measured using an SI-traceable caliper. During LDA calibrations, the chopper was supported by remotely-controlled translation stages. (See figures 1 and 2) The rim of the chopper was systematically moved through the intersection laser beams (i.e. the LDA’s sensing volume). Slight blemishes on the rim of the disk served effectively as tracer particles that provided adequate scattering signals which the LDA converted to velocities.

In section 2, we describe the chopper standard. Section 3 presents the model for calibrations. Section 4 describes the calibration procedure and displays typical calibration data. We calibrated *in situ* two NIST-owned LDAs; one $\left({LDA}_{\text{roof}}\right)$ was mounted on the roof of NIST’s wind tunnel; the other $\left({LDA}_{\text{wall}}\right)$ was mounted on a wall of the wind tunnel. In section 5 we test the consistency of the two calibrations in the wind tunnel. An uncertainty analysis for the calibration of each of these LDAs is provided in section 6.

## 2. The chopper standard

We used the periphery of an optical chopper blade (Thorlabs Model MC1F10HP)^2^ as a velocity standard. During a calibration, the chopper was mounted on (X,Y,Z) translation stages. (See figure 1.) The stages were oriented so that the blade’s plane coincided with the plane of the LDA’s intersecting laser beams. (See figure 2.) As manufactured, the blade’s diameter and thickness were 101.6 mm and 0.254 mm. This thickness was less than the 0.3 mm diameter of the sensing volume of ${LDA}_{\text{roof}}$. If this blade were used for calibrating ${LDA}_{\text{roof}}$, the blade would have to be scanned parallel to its rotation axis through the LDA’s sensing volume while integrating the velocity signal from LDA and accounting for scattering from the blade’s corners. To avoid these complications, we attached a rim to the blade, effectively increasing its width to 2 mm. The rim was a hard, black acetal plastic ring that had an L-shaped cross section. The shorter leg of the L functioned as the rim of the blade; it was 2.0 mm wide and it added 1.0 mm to the diameter of the blade. The longer leg of the L extended approximately 4 mm along the side of the blade where it was epoxied to the blade.

After the rim was installed, we measured the diameter of the blade using a digital caliper (Mitutoyo Model CD-12” CPW, SN 1003160)^2^ that had been recalibrated at NIST. The average and standard deviation of 10 diameter measurements at intervals of 36° was 103.721 × (1 ± 0.051%) mm. The uncertainty of the caliper contributed the additional fractional uncertainty 0.056% to the blade’s diameter. The ambient temperature during measurements of the blade’s diameter and during its use for LDA calibrations was (21 ± 1) °C. In this range, the thermal expansion of the chopper (steel blade + rim) is less than 1/10th of the uncertainty of the blade’s diameter; therefore, we ignore it.

The rotation of the chopper blade was controlled by the manufacturer’s Optical Chopper System (Thorlabs Model MC2000B)^2^. The system generates 100 pulses/revolution that we counted to determine the blade’s angular velocity. Using 2 s integration times, we measured fractional frequency instabilities on the order of 10^−5^ or less. This contributed 0.0005% to the k=2 uncertainty to row 2 of table 2.

The chopper was mounted on a stack of three motorized, linear, translation stages. (Standa Ltd, Vilnius, Lithuania, part number 8MT175–150XYZ)^2^ After the chopper blade was aligned, the stack translated the blade parallel to the X-,Y-,Z- axes in steps of 2.5 μm under computer control.

## 3. Geometry-based calibration model

Because the fringe spacing is not uniform, the calibration factor of an LDA must be determined by a volume-weighted average over the entire fringe pattern (sensing volume). Previous implementations of volume averaging [2, 4, 5, 11] required precise positioning of the chopper’s rim in the sensing volume. To relax this requirement, we developed a model that uses data taken without absolute positioning of the chopper’s rim. Then, we determined both the absolute position and the calibration coefficient by fitting the model to the data.

For LDA calibrations, the chopper is mounted on its stages, placed in the wind tunnel, and aligned with the LDA. This *in situ* calibration avoids dismounting and remounting the LDA. The disk rotates at a constant speed and traverses in the x-z-plane while the LDA gathers data. (See figure 2 for coordinates.)

**Figure 3.**
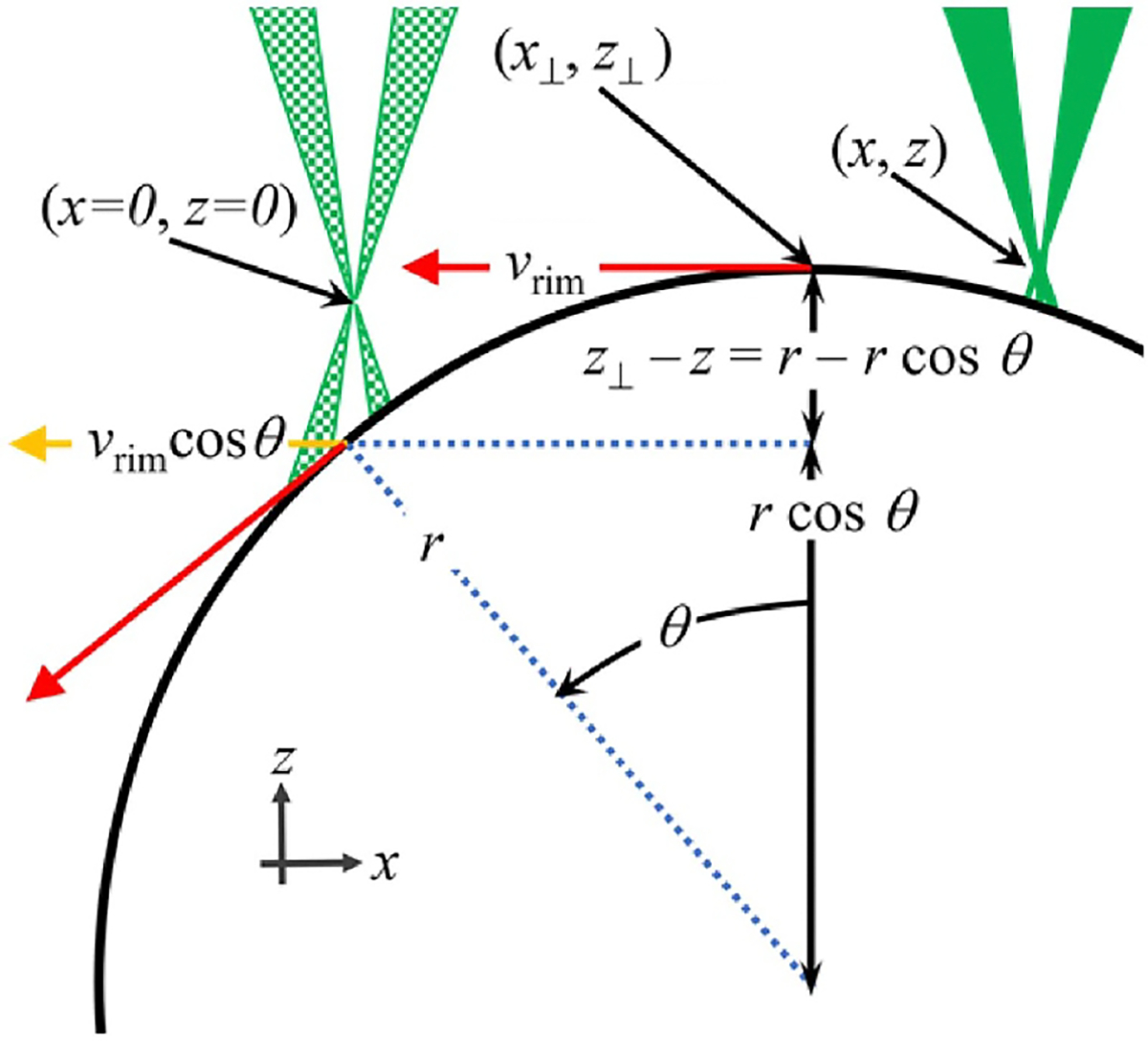
The geometry of an X-scan. The solid green triangles represent the intersecting laser beams that scatter off the chopper’s rim at the point defined as (x=0,z=0). After the chopper is translated in the x-direction, the laser beams (checked green triangles) scatter from the point (x,y,z). Throughout the x scan, the LDA measures the $v_{\text{rim}}$, that is $v_{\text{rim}}cos(\theta )$.

To derive a model for the calibration factor, we consider how the velocity measured by the LDA, $v_{\text{LDA}}$, varies as a function of x and z. The variation has two dominant sources: (1) misalignment of the tangential velocity of the disk with the plane of the LDA’s fringes, and (2) the change in the distance between the rim of the disk and the intersection of the laser beams. (This distance is measured as a function of the position along the X-axis.) The LDA measures $v_{\text{rim}}\;cos\theta$, the component of the rim’s velocity that is perpendicular to the fringes (see figure 3). The calibration factor C is:

(1) $$Cv_{LDA}=v_{rim}cos\theta =v_{rim}\sqrt{1-{\left(\frac{x\;-\;x_{⊥}}{r}\right)}^{2}}$$

where r is the radius of the disk and $x_{⊥}$ is the x coordinate associated with the intersection of the rim the bisection of the laser beams when the bisection is passed though the center of rotation of the disk.

Because the calibration factor depends on the fringe pattern’s spacing, gradients in the spacing introduce spatial dependences of C (i.e. C=C(x,y,z)). The variation of the fringe spacing along the x-direction is fully sampled by tracer particles passing through the sensing volume; therefore, the x-dependence of C is neglected in this discussion. By translating the disk along the Y-axis, we measured the y-dependence of C(x,y,z). The average value dC/dy=0.06%/mm. Thus, the rim’s departure from a perfect right circular cylinder has a small conical component; this is accounted for by the entry 0.009 in row 6 of the uncertainty budget. Along the z-direction dC/dz was as large as 1.35 %/mm; therefore, it must be carefully characterized.

We represent the z-dependence of the calibration factor by the polynomial:

(2) $$C\left(x,z\right)=\frac{v_{rim}}{v_{LDA}}\sqrt{1-{\left(\frac{x\;-\;x_{⊥}}{r}\right)}^{2}}\sum_{n=0}^{N}c_{n}z_{eff}^{n},$$

where $\left\{c_{n}\right\}$ are the coefficients of the $n^{\text{th}}$ order correction to the fringe spacing, N is the order the approximation, and $z_{\text{eff}}$ is the height of the center of the sensing volume above disk’s rim. As shown in figure 3, when the disk translates in the x-direction, the effective z-position changes as

(3) $$z_{eff}=z-z_{⊥}+r-\sqrt{r^{2}-{\left(x-x_{⊥}\right)}^{2}}.$$

Combining equations (2) and (3) gives the full expression for the calibration factor:

(4) $$C(x,z)=\frac{v_{\text{rim}}}{v_{LDA}}\sqrt{1-{\left(\frac{x\;-\;x_{⊥}}{r}\right)}^{2}\sum_{n=0}^{N}c_{n}}\;\;\times {\left(z-z_{⊥}+r-\sqrt{r^{2}-{\left(x-x_{⊥}\right)}^{2}}\right)}^{n}.$$

We fit equation (4) to data for $v_{\text{rim}}$ and $v_{\text{LDA}}$ taken at various x- and z-positions using weighted least-squares. The LDA’s counting rate is proportional to the probability of a tracer particle traversing the sensing volume; therefore, we use the counting-rate (see figure 5) at each location as a weight factor w(x,z)=counts(x,z)/(totalcounts) for the least-squares fit. This fit allowed us to determine the optimal values of $x_{⊥},\;z_{⊥}$, and $\left\{c_{n}\right\}$, making precise alignment along the x- and z-axes unnecessary. Since the position of a particle passing through the sensing volume is not known, the calibration factor needs to be independent of position. This is achieved by using the average calibration factor around the point ($x=x_{⊥},\;z=z_{⊥}$). The average $\langle C(x,z)\rangle_{x,z}\approx c_{0}+O\left(x^{2}\right)+O\left(z^{2}\right)$, as the anti-symmetric, linear term contributes exactly zero. For both LDAs, we used N=3; increasing N above 3 did not change $c_{0}$. There were 21 measurement locations along the z direction and 20 points along the x for a total of 420 measurement locations. The covariance of the fit for $c_{0}$ was, typically, 0.000 59; this contributed 0.058% to the k=2 uncertainty on row 4 of table 2.

## 4. Calibration procedure

Table 1 lists the properties of each LDA that we calibrated.

Each *in situ* calibration began by placing the spinning disk calibrator in the wind tunnel and aligning it with the LDA as sketched in figure 2. After the disk was aligned, it rotated at a constant speed while it was translated in the X-Z-plane and the LDA’s measurements of $V_{\text{rim}}(x,\;z)$ were recorded.

**Figure 4.**
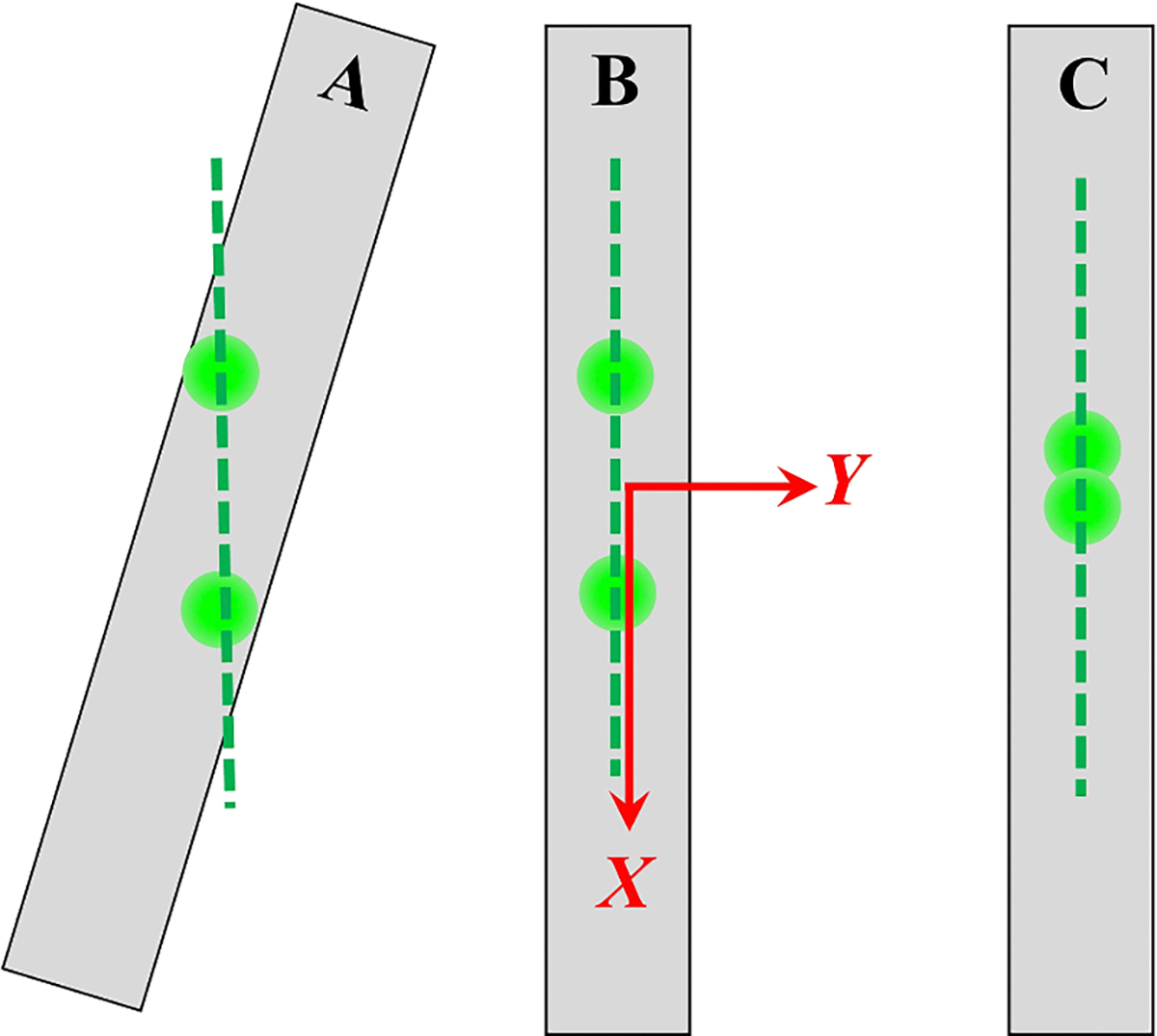
Schematic views of the chopper blade viewed on edge during its alignment with an LDA. (A): The blade must be rotated about the Z-axis until it is parallel to the plane of the laser beams (green dashed line). (B): The planes are aligned; however, the blade must be raised or lowered until the laser beams intersect on the blade’s rim. (C): The blade is aligned and close to the correct height. We identify this location with the coordinates (x=0,y=0,z=0).

In preparation for calibrating an LDA, we reduced the laser power to an eye-safe level and placed the chopper (while mounted on x-,y-,z- translation stages) on the floor of the wind tunnel. We positioned the chopper’s rim so that the LDA laser beams formed two, well-separated, bright spots on the rim. (See figure 4.) Then, we rotated the chopper about the Z-axis until the plane of the chopper’s blade was parallel to the plane of the intersecting laser beams, as judged by eye. A large separation between the spots facilitates precise alignment. This procedure produces an alignment accurate to within the spot diameter divided by the separation distance. The uncertainty of this alignment is accounted for by the entry 0.022 on row 8 of table 2.

After the plane of the chopper’s disk was parallel to the plane of the LDA, the chopper’s Z-axis stage was made parallel to the plane of the LDA. This was achieved by manipulating its screw adjustable feet such that the laser spots remain on the rim of the chopper as it is traversed in the z-direction. This final alignment does not effect the tangential velocity’s alignment with the LDA; however, it ensures that the laser beams’ intersection remains on the rim while the chopper is scanned through the sensitive volume in the z-direction.

**Figure 5.**
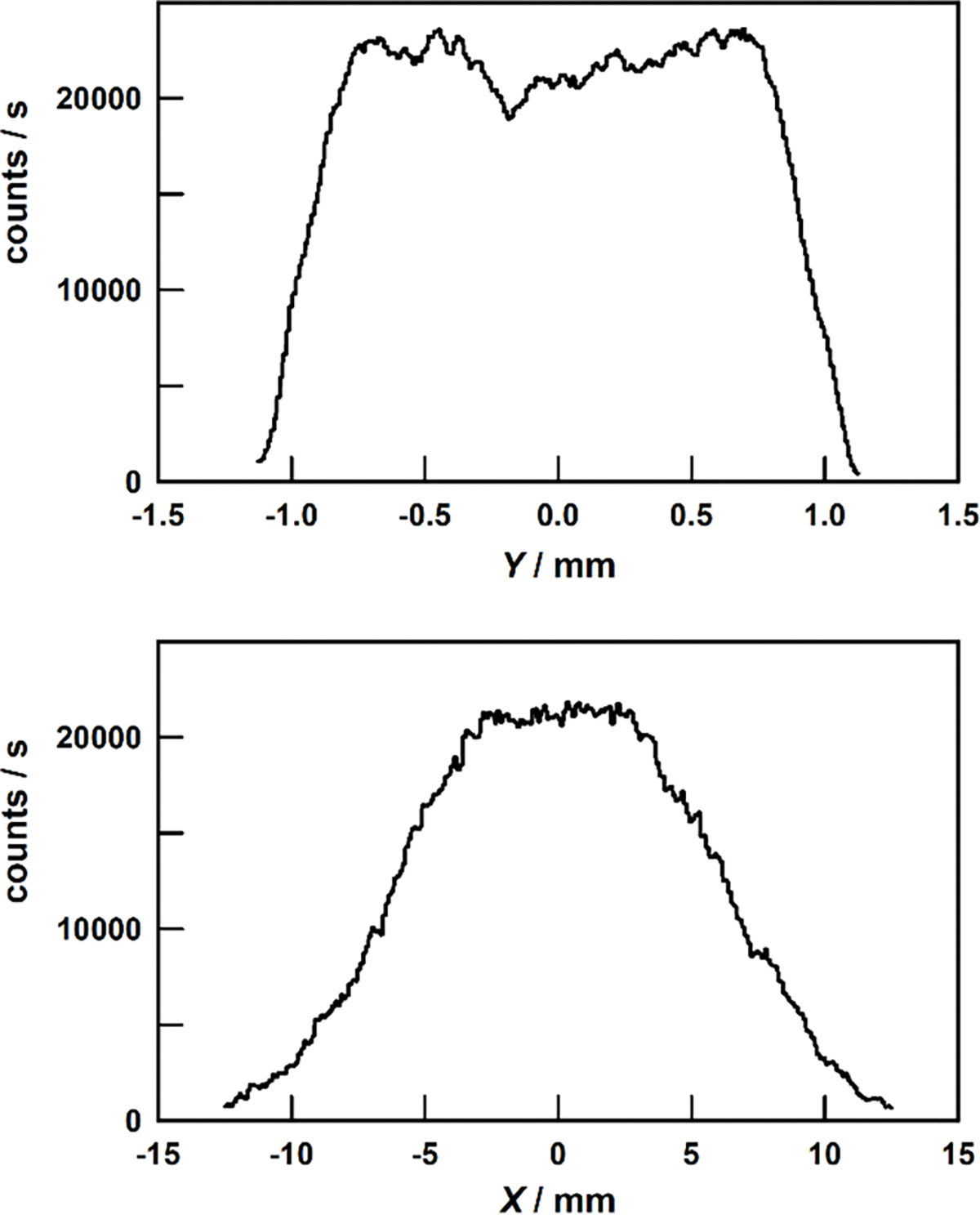
Top: Counting rates from ${LDA}_{\text{roof}}$ during scans parallel to the Y-axis (top) and to the X-axis (bottom). For both plots, the data were averaged over 0.05 mm-wide Y intervals.

To center the blade’s rim under the LDA’s sensitive volume, the chopper is first stepped parallel to Y-axis and then parallel to the X-axis while recording the LDA’s counting-rate. The counting-rate falls abruptly as the rim is moved in the y-direction out of the sensitive volume. (See figure 5.) We defined the location y=0 as the ‘middle’ of the counting-rate peak (figure 5, top) by calculating its median y-position using the equation:

$$\int_{-1.125\;mm}^{\text{middle}}(\text{count}\;/s)\;dy=\int_{\text{middle}}^{1.125\;mm}(\text{count}\;/s)\;dy.$$

Across the peak, |y|<0.7mm, the counting rate varied by approximately 25%, probably because the reflectivity of the rim was not uniform. Outside of the range |y|<0.7mm, the counting rate rapidly decreased to zero as parts of the 0.3 mm-wide laser beams began to pass beside the 2 mm-wide rim.

We calibrated two LDAs in NIST’s wind tunnel following two different procedures: (1) the ‘surface fit method’ discussed above, and (2) the ‘weighted means’ procedure described by [2, 4, 5].

**Figure 6.**
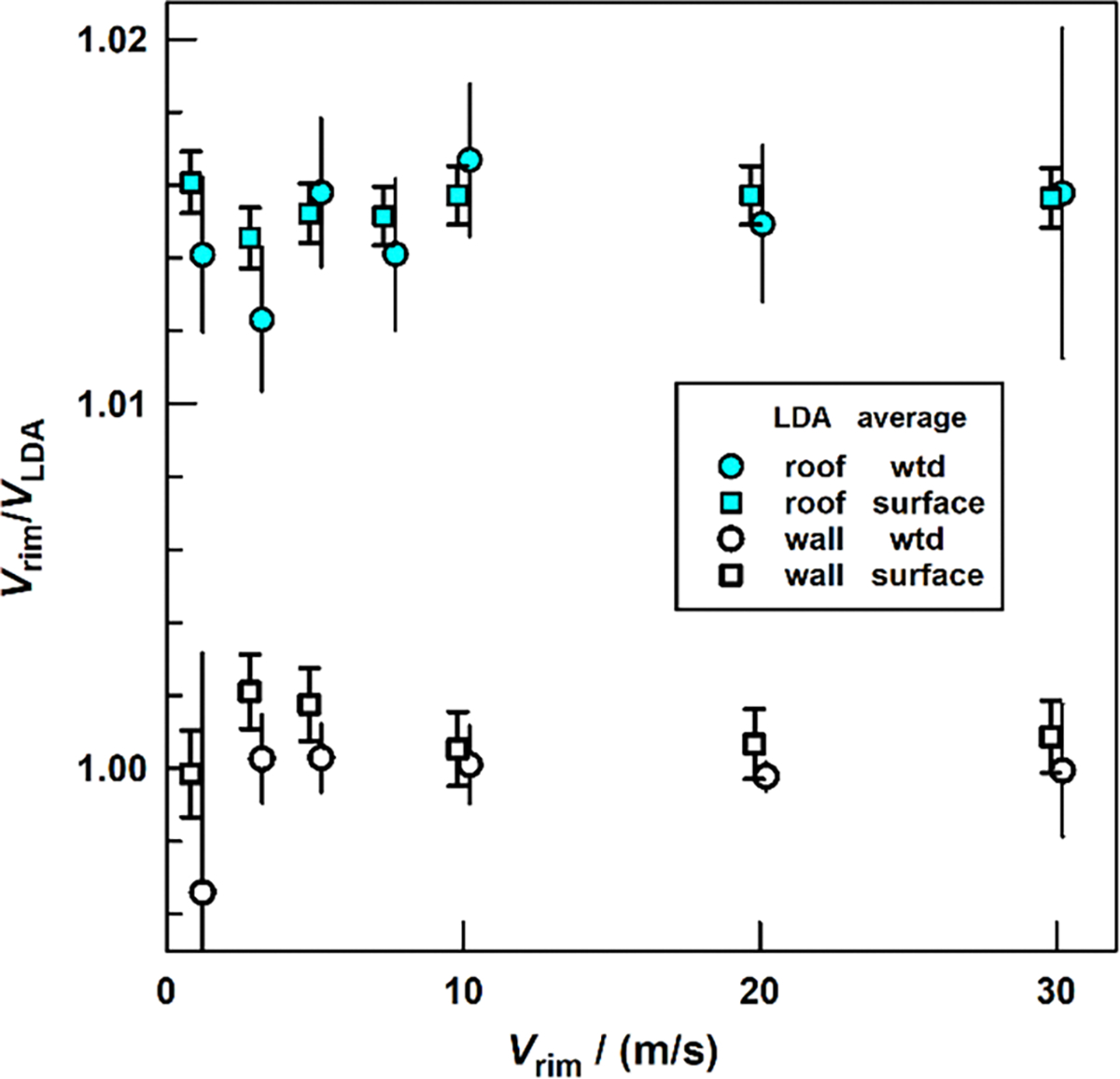
Calibration factors determined by weighted average method [2] and by surface fit (this work).

The calibration results at seven air speeds between 1 m s^−1^ and 30 m s^−1^ are displayed in figure 6. Figure 6 does not show any obvious air speed dependence of the calibration factors. Both calibration protocols gave mutually consistent results; however, the surface fit method produces smaller uncertainties.

## 5. Consistency tests

The low uncertainty of each calibration in figure 6 is evidence that the chopper velocity standard and both calibrated LDAs are precise and stable. To search for possible biases in these instruments and protocols, we tested the mutual consistency of air speed measurements made in the wind tunnel using both LDAs.

Conceptually, the simplest consistency test would use both LDAs to measure the identical flow simultaneously. To do this, the LDAs would have to be reinstalled so that their sensitive volumes overlapped and their fringe patterns were parallel. However, reinstalling the LDAs might misalign the LDAs’ optics and certainly would change the effects of the windows that admit the laser beams into the wind tunnel.

Instead of reinstalling the LDAs, we compared their performances using transfer standards. The transfer standards were two nearly-identical Pitot tubes with extensive calibration histories. (We labeled these Pitot tubes: ‘Pitot, blue’ and ‘Pitot, red’)

The comparison was done while the LDAs were in their normal wall and roof locations and their sensitive volumes were located in the same wind tunnel cross section, but 0.5 m apart in the horizontal direction. Unlike the chopper-based calibration, the comparison was sensitive to the orientation of the LDAs with respect to the flow in the wind tunnel. When ${LDA}_{\text{roof}}$ was installed, it was aligned with respect to the wind tunnel’s wall and floor to within ±1°. In prior tests spanning the range (1–30) m s^−1^, the horizontal component of the wind velocity was measured to be within ±1° of the tunnel’s axis. We assumed that the same was true for the vertical component. Because $V_{\text{LDA,roof}}$ depends on the cosine of the misalignment angle with the flow direction, the angle uncertainty has negligible consequences. After calibrating ${LDA}_{\text{wall}}$ with the chopper, we rotated it until the plane of its crossing laser beams was horizontal, as determined using an inclinometer. To align ${LDA}_{\text{wall}}$ in the horizontal plane, we established a steady flow inside the tunnel and rotated ${LDA}_{\text{wall}}$ to maximize its measurement of the horizontal component of the air speed. We estimate that the precision of this alignment was ±1 °.

**Figure 7.**
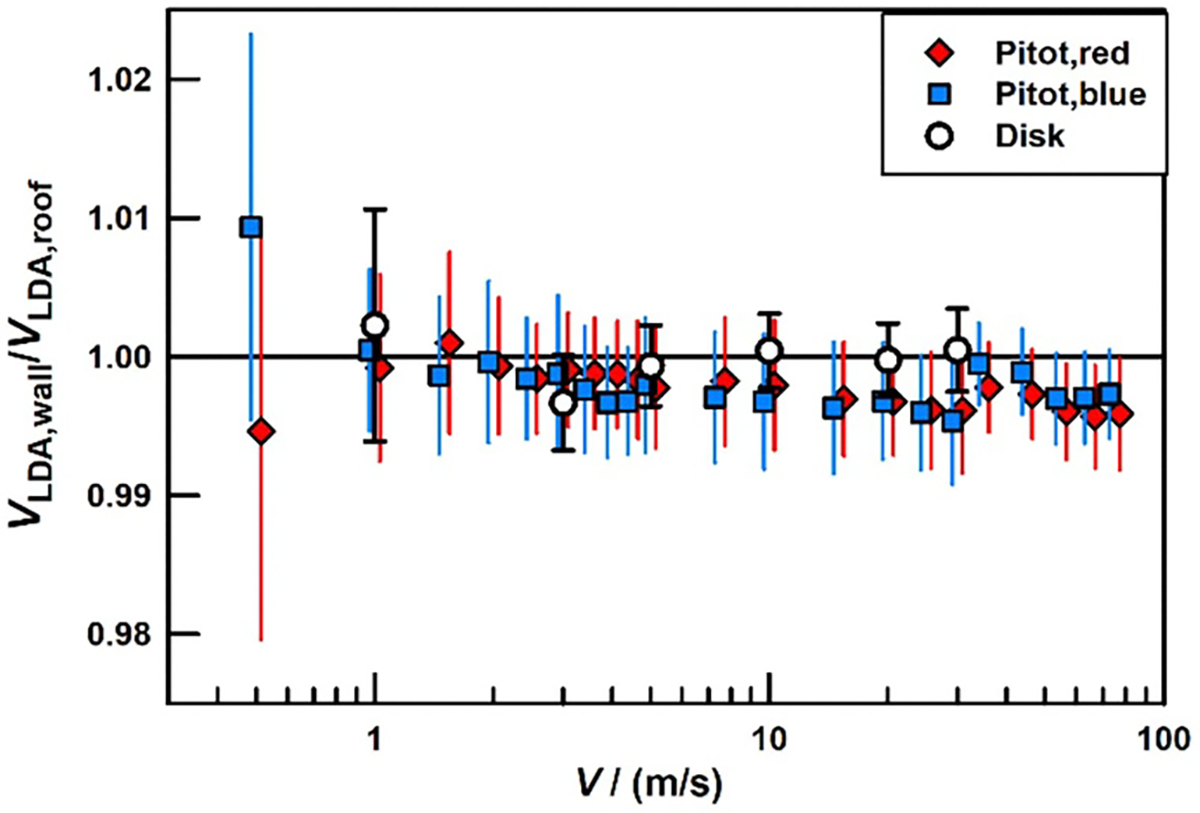
Comparison of two LDA calibrations as a function of air speed or disk speed. The filled symbols result from LDA measurements of air speed using Pitot tubes as transfer standards. The open circles compare the LDAs using data only from the spinning disk.

Each Pitot tube was placed 16 cm downstream of an LDA’s sensing volume to minimize blockage effects [12]. The readings from the LDA and Pitot tube were recorded and used to calculate the ratios $V_{\text{LDA,roof}}/V_{\text{pitot,red}}$ and $V_{\text{LDA,wall}}/V_{\text{pitot,blue}}$ as a function of the air speed. Then, the positions of the Pitot tubes were exchanged to measure the ratios $V_{\text{LDA,roof}}/V_{\text{pitot,blue}}$ and $V_{LDA,\text{wall}}/V_{\text{pitot,red}}$ at nearly the same air speeds. We divided the appropriate pairs of these ratios to obtain two independent values of $V_{\text{LDAroof}}/V_{\text{LDAwall}}$. The results are shown in figure 7. For this comparison, the calibration factors of the Pitot tubes cancel out of the ratio of ratios. Therefore, the Pitot tubes may be uncalibrated, but they must be stable, and the differential pressure gages used with the Pitot tubes must be stable and linear. Also, the Pitot tubes must be aligned with the axis of the wind tunnel.

The independent comparisons performed with the two Pitot tubes shown in figure 7 are in excellent mutual agreement. In both experiments, the 95% confidence interval has large overlap with the ideal ratio of 1. However, Pitot tube comparison has a trend toward values less than 1.00 as the air speed increases. This trend is similar in the comparisons using the ‘red’ and ‘blue’ Pitot tubes. Thus, it is unlikely that the trend resulted from misaligning the Pitot tubes when they were installed in the two different locations 0.5 m apart. (Each Pitot tube was aligned with the wind tunnel’s walls by eye.) The trend reaches 0.2% at 75 m s^−1^.

Such a 0.2% difference at 75 m s^−1^ would occur if the wind flow vector near ${LDA}_{\text{roof}}$ were parallel to the axis of the wind tunnel while the wind flow vector near ${LDA}_{\text{wall}}$ was 3.6° off the axis. (This is a subject for future study.) Whatever the trend’s origin, its effect is small compared to the 0.63% uncertainty of each Pitot tube comparison.

We also compared the LDA calibrations using the data acquired while each LDA was calibrated with the spinning disk. We formed the ratios $R_{\text{roof}}\equiv \left(C_{\text{roof}\;}V_{LDA,\text{roof}}\right)/V_{\text{rim}}$ and $R_{\text{wall}}\equiv \left(C_{\text{wall}}\;V_{LDA,\text{wall}}\right)/V_{\text{rim}}$, where each C is the calibration coefficient in equation (1). The ratio $R_{\text{roof/}}/R_{\text{wall}}$ does not depend upon air speed measurements; it does test the effectiveness of the calibration protocol in sampling the sensitive volumes of the two LDAs. The ratio $R_{\text{roof/}}/R_{\text{wall}}$ is plotted as open circles in figure 7. Within the uncertainties, $R_{\text{roof/}}/R_{\text{wall}}=1.00$, independent of the disk’s speed throughout the range (1–30) m s^−1^.

## 6. Uncertainty analysis

The dominant uncertainty sources of the LDA calibrations result from (1) measuring the chopper’s diameter, and (2) averaging over the sensitive volume. The k=2, uncertainty of the chopper’s angular frequency was negligible; it was only 0.0005% as measured using an SI-traceable frequency counter. (Table 2, row 2.)

We traced $V_{\text{rim}}$ to the SI units of length and time by measuring the disk’s diameter and its angular frequency. We used a caliper to measure the disk’s diameter. The caliper was compared to a reference gage block that had been calibrated by NIST’s Dimensional Metrology Group. Multiple caliper measurements at 10 different angles were fitted by an ellipse with major and minor axes of 103.78 mm and 103.67 mm. The k=2 uncertainty of the average diameter from the fit was 0.016 mm, equivalent to 0.015% of the diameter. The uncertainty in the calipers used to determine the diameter is 0.06 mm (0.058 %) at the 95% confidence interval (k=2). (Table 2, row 1) Auxiliary measurements using an optical coordinate measuring machine (OCMM) provided 1008 coordinate (x,y) pairs that determined the shape of the rim in the plane bisecting the rim’s thickness. The pairs were fitted by circles to determine a root-mean-square diameter $D_{\text{RMS}}=103.700\;mm$ and the diameters $D_{\text{max}}$ and $D_{\text{min}}$ of bounding circles. Ratios of these diameters were $D_{\text{max}}/D_{RMS}=1.002\;32$ and $D_{\text{min}}/D_{RMS}=0.998\;54$. The OCMM measurements confirmed that the caliper measurements were sufficiently detailed to obtain an accurate RMS diameter.

We computed the tangential velocity from the rim’s diameter $D_{\text{RMS}\;}$ and rotation frequency f using $v_{\text{rim}}=\pi D_{\text{RMS}}f$. If the rim’s rotation axis were displaced from the rim’s geometric center by a small distance δ, the rim’s effective diameter $D_{\text{effective}}$ would be approximately reduced by the quantity $\left(1-\delta^{2}/D_{\text{RMS}}^{2}\right)$. Thus, a 1 mm displacement would generate a negligible 0.005% reduction of $v_{\text{rim}}$.

During the diameter measurements and the present LDA calibrations, the temperature of the disk was in the range (21 ± 1) °C. The thermal expansivity of typical acetal plastics is in the range (8–12) × 10^−5^ K^−1^. [13] The thermal expansivity of steels is much smaller, on the order of (1–2) × 10^−5^ K^−1^. We made two conservative assumptions: (1) the thermal expansion of the diameter of the {disk+rim} has the expansivity of the acetal plastic, and (2) that future calibrations are subject to a k=2 temperature uncertainty in the range (21 ± 3) °C. These assumptions add an uncertainty to the diameter of less than 0.036% with k=2. (Table 2, row 3)

The calibration factor, $c_{0}$, is determined from a surface fit weighted by the data counts and its uncertainty is estimated from the covariance of the fit; the uncertainty reported in table 2, row 4 is a typical value.

The combination of all these sources of uncertainty are shown in table 1 and are estimated to be 0.094% at the (k=2) 95% confidence interval.

## Figures and Tables

**Table 1.** Properties of calibrated lasers.

Mounting	Roof	Wall

Manufacturer	Dantec^2^	Artium^2^
Wavelength	513.5 nm	532 nm
Focal length	1.2 m	0.35 m
Sensing volume length	9.9 mm	0.89 mm
Sensing volume diameter	0.3 mm	0.1 mm
Fringe spacing	8.5 μm	2.34 μm

**Table 2.** Type A and type B uncertainties of calibration at 95% confidence level.

	Sources	Value	*U*_A_/%	*U*_B_/%

1	Disk Diameter (caliper)	103.721 mm	0.015	0.058
2	Disk speed (frequency counter)		0.0005	
3	Disk’s temperature	±3 °C		0.036
4	Fit calibration factor (1–30) m s^−1^	1.015	0.058	
5	Misalignment of disk and *X*-axis			0^a^
6	Misalignment of disk and *Y*-axis			0.009
7	Misalignment of disk and *Z*-axis			0^a^
8	Misalignment of disk with beam plane			0.022

	**Root sum squares, (*k* = 2)**			**0.094**

a Free Parameter in fitting equation (4).

## Footnotes

1 Unless otherwise stated, all uncertainties reported in this document are expanded standard uncertainties with coverage factor k=2 corresponding to a 95% confidence interval.

2 In order to describe materials and procedures adequately, it is occasionally necessary to identify commercial products by manufacturer’s name or label. In no instance does such identification imply endorsement by the National Institute of Standards and Technology, nor does it imply that the particular product or equipment is necessarily the best available for the purpose.
